# *Schaalia turicensis* endometritis in a diabetic patient: MALDI-TOF MS overcomes diagnostic barriers in a non–IUD-associated case

**DOI:** 10.3389/fmed.2025.1593385

**Published:** 2025-08-05

**Authors:** Yan Liu, Mei Li, Yunyun Ding, Yong Gao, Tuantuan Li, Xiaowu Wang

**Affiliations:** ^1^Department of Clinical Laboratory, The Second People’s Hospital of Fuyang City, Fuyang, China; ^2^Fuyang Infection Disease Clinical College of Anhui Medical University, Anhui, China

**Keywords:** endometritis, *Schaalia turicensis*, microbial identification, treatment regimen, case report

## Abstract

*Schaalia (Formerly Actinomyces) turicensis* is a single-cell prokaryotic microorganism and a common component of the normal human oral flora. If it enters other parts and causes infection, it may lead to actinomycosis. We present the case of a 74-year-old female patient from Fuyang who developed bacterial endometritis due to *Schaalia turicensis*. The bacterial culture of painless cervical dilation with drainage and curettage showed slow-growing, Gram-positive rods, which were identified by matrix-assisted laser desorption/ionization time-of-flight mass spectrometry (MALDI-TOF MS) as *Schaalia turicensis*. The patient was initially treated empirically with cefathiamidine and tinidazole for 3 days, with no improvement of symptoms. Subsequently, the treatment regimen was adjusted to intravenous infusion of amoxicillin and clavulanate potassium (1.2 g) every 8 h for 7 days. Resulted in significant alleviation of the patient’s symptoms. Clinicians should be aware of other types of infections that may be caused by *Schaalia turicensis*.

## 1 Introduction

Chronic endometritis (CE) is a chronic inflammation that persists in the endometrial stroma, with histological findings revealing plasma cell infiltration (1). The clinical symptoms of CE are non-specific and may manifest as atypical bleeding, dysmenorrhea, abnormal vaginal discharge, and dyspareunia (2, 3). These conditions are often not identifiable through routine diagnostic tests and may be easily overlooked by physicians. The pathogenesis of CE is complex and diverse, among which bacterial infection is the most common cause. The main pathogens include *Streptococcus* (27%), *Escherichia coli* (11%), *Enterococcus faecalis* (14%), and *Ureaplasma urealyticum* (11%) (4). Clinical infections caused by *Schaalia turicensis* (previously *Actinomyces turicensis*) are rare. *Schaalia turicensis*, formerly known as *Actinomyces turicensis*, is an anaerobic or microaerophilic Gram-positive non-acid-fast bacillus. It was originally classified within the genus *Actinomyces* (5, 6). The identification of this type of infection in clinical practice is challenging due to the extended culture times required for detection. Additionally, the lack of expertise in using specialized equipment and limited access to such resources further complicates the diagnostic process (7).

We report a case of a 74-year-old female patient who was admitted for treatment due to abnormal vaginal bleeding, foul-smelling discharge, and a suspected uterine mass detected by ultrasound. The patient underwent a curettage procedure to obtain purulent secretions and tissue samples, which were subsequently subjected to microbiological and histopathological examinations. Histopathological examination did not reveal any cancer cells, while the microbial culture colonies were identified as an infection with *Schaalia turicensis* through matrix-assisted laser desorption/ionization time-of-flight mass spectrometry (MALDI-TOF MS).

## 2 Case presentation

### 2.1 Case description

**FIGURE 1:**
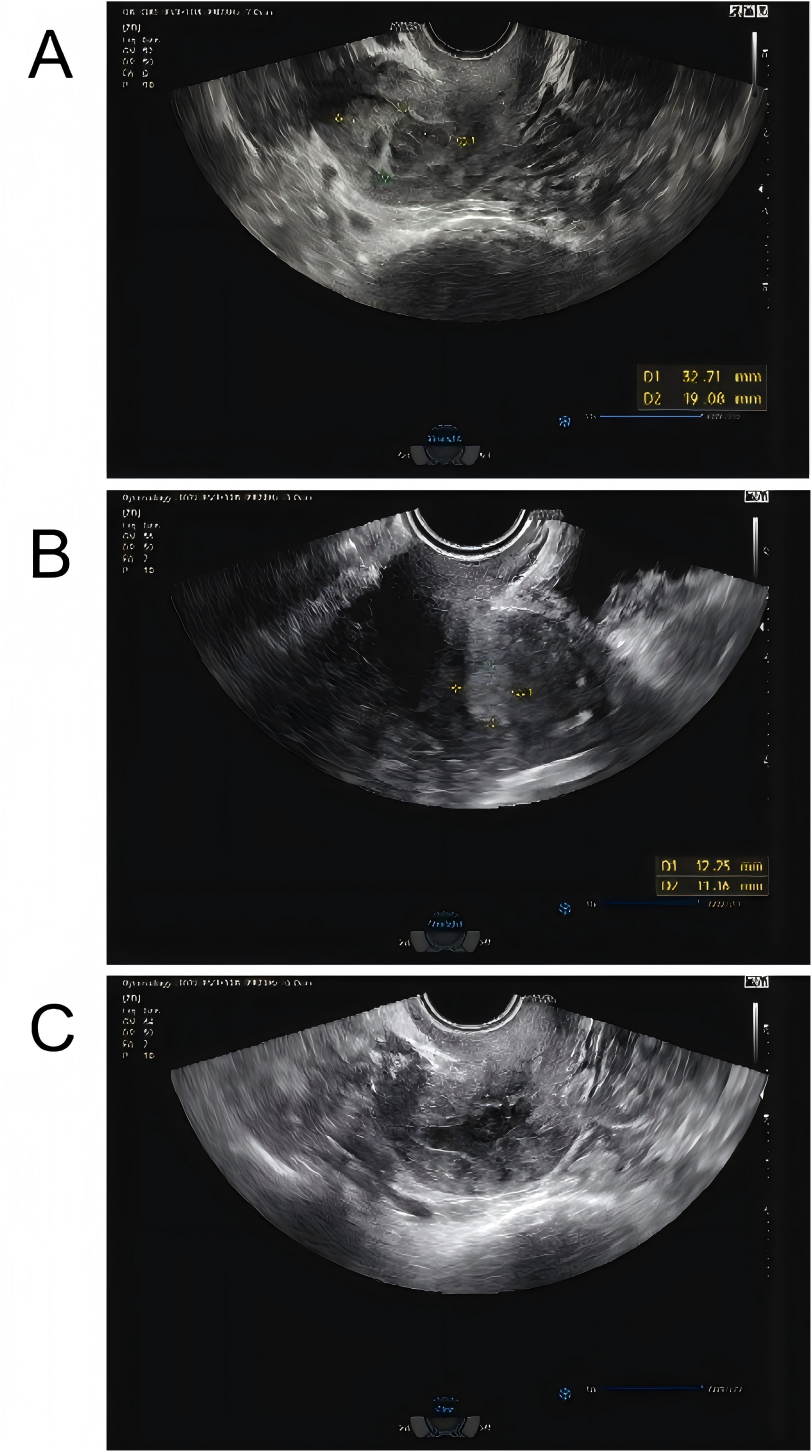
**(A)** Color Doppler ultrasound showed a mixed echoic mass in the uterine cavity initially, measuring approximately 33 mm × 19 mm, with patchy strong echoes visible at the edges. **(B)** Reexamination using color flow Doppler ultrasound showed that a mixed echoic mass in the uterine cavity, measuring approximately 12 mm × 13 mm × 11 mm, which was smaller than the previous lesion. **(C)** One month later, and the patient had an ultrasound, reporting that the uterine lesion had completely absorbed.

The patient is a 74-year-old woman who has been naturally menopausal for over 20 years. She has no history of smoking or alcohol consumption. Her obstetric history is gravida 2, para 2 (G2P2), with no history of adverse pregnancy outcomes. The gynecological examination revealed the following findings: the vulva was of senile type; the vagina was patent with grayish-pink discharge; the cervix had mild erosion and bled on contact; the uterus was atrophied and non-tender; and the adnexa and parametria showed no abnormalities. She has had diabetes for 20 years, with poor blood glucose control. During this period, she had not experienced any vaginal bleeding or discharge. However, in October 2024, she began to experience vaginal bleeding without any obvious cause. The bleeding was minimal, bright red, and occasionally accompanied by blood clots, though she did not experience lower abdominal pain. In December 2024, a color Doppler ultrasound performed at a local health clinic revealed an intrauterine space-occupying lesion. However, the patient did not seek further treatment but self-administered antibiotics (she could not recall the specific antibiotics) for 2 weeks. Since her symptoms did not improve, she did not pay sufficient attention to them. By January 2025, the amount of vaginal bleeding had decreased, but she began to experience a yellowish, watery discharge. She then came to our hospital for treatment. A color Doppler ultrasound showed a mixed echoic mass in the uterine cavity, measuring approximately 33 mm × 19 mm (Figure 1A, with patchy strong echoes visible at the edges. Based on these findings, the outpatient department preliminarily diagnosed her with “uterine space-occupying lesion” and decided to admit her for inpatient treatment.

**FIGURE 2:**
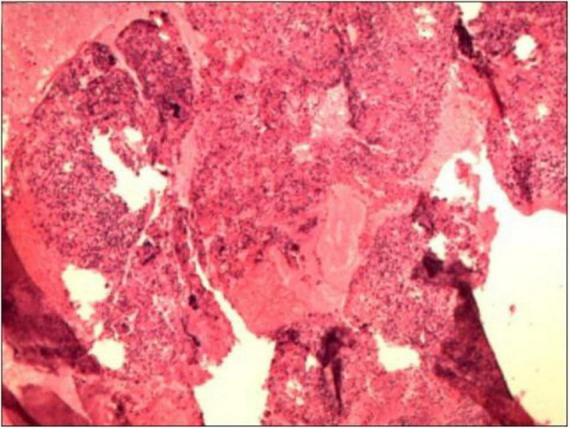
The histopathological examination of the submitted (endometrial) material revealed: fragments of endometrium admixed with significant amounts of inflammatory exudate and necrotic debris.

Routine examination of vaginal discharge shows an increase in the number of white blood cells and a decrease in cleanliness. The results of the urine tests and recent routine blood examination were normal. The fasting blood glucose level reached 8.9 mmol/L, and the blood glucose level 2 h after a meal reached 24.7 mmol/L. The patient had a white blood cell count of 5.89 × 109 cells/L in a recent routine blood examination and cancer antigen 125 (CA125) 13.23 U/mL. Purulent discharge was collected from the patient during a painless cervical dilation and drainage procedure, as well as a curettage surgery. Purulent discharge was initially dark and foul-smelling, later becoming yellowish and watery. The purulent discharge was sent for bacterial culture, and the curettage specimens were analyzed pathologically. Anti-inflammatory treatment was administered empirically by intravenous injection of cefathiamidine and tinidazole for 3 days. The patient was followed up 3 days after the last anti-inflammatory treatment, but her discomfort symptoms had not eased. The histopathological examination of the submitted (endometrial) material revealed: fragments of endometrium admixed with significant amounts of inflammatory exudate and necrotic debris (Figure 2). The culture of the purulent discharge, which was incubated for 5 days, reported the presence of *Schaalia turicensis*. The pathological analysis of the curettage specimens revealed no cancerous cells. Subsequently, based on microbial identification and antimicrobial susceptibility testing, the treatment regimen was adjusted to intravenous infusion of Amoxicillin and Clavulanate Potassium (1.2 g) every 8 h for 7 days. Reexamination using color flow Doppler ultrasound showed that a mixed echoic mass in the uterine cavity, measuring approximately 12 mm × 13 mm × 11 mm (Figure 1B, which was smaller than the previous lesion. The patient received the adjusted antibiotic treatment as prescribed. Her symptoms gradually improved, with the yellowish, watery discharge decreasing in quantity. Further evaluation of the patient’s condition was conducted. The patient’s vital signs were stable, and there were no significant abnormalities in her physical examination. Based on the patient’s good condition, the patient was discharged and prescribed oral amoxicillin (0.5 g q8h) for 1 month. One month later, a telephone follow-up was conducted, and the patient had an ultrasound at the local health hospital, reporting that the uterine lesion had completely absorbed (Figure 1C).

### 2.2 Bacterial culture, identification, and susceptibility testing

#### 2.2.1 Culture and morphological identification

The pus specimens were cultured aerobically and anaerobically on Columbia blood agar plates. Aerobic cultures were incubated at 35°C in 5% CO_2_. Anaerobic cultures were incubated at 35°C using an anaerobic bag system with a gas generator. Colony growth was monitored over 48–72 h. Gram staining was performed on isolates from positive cultures for microscopic examination.

#### 2.2.2 Bacterial identification

Bacterial morphology was observed under a microscope using Gram staining (BASO Gram Stain, rapid method). Identification was performed using a MALDI-TOF MS.

#### 2.2.3 Antimicrobial susceptibility testing

CLSI M45 defines “Infrequently Isolated/Fastidious Bacteria” as pathogens with lower infection frequency compared to microorganisms in CLSI M02/M07/M100. Key examples comprise coryneform bacteria, *Bacillus* spp., *Granulicatella* spp., *Aeromonas* spp., and certain bioterrorism-related agents. CLSI M45-A3 provides specific breakpoints and methodologies tailored for testing such organisms, including coryneform bacteria and related genera. *Schaalia turicensis* is closely related to the genus *Corynebacterium*. The antimicrobial susceptibility testing was conducted according to the instructions provided with the *Corynebacterium* susceptibility kit. The interpretation of susceptibility results was based on the M45 A3 criteria for *Corynebacterium* established by the Clinical and Laboratory Standards Institute (CLSI) (8). The quality control strains used were *Escherichia coli* ATCC 25922 (for gentamicin) and *Streptococcus pneumoniae* ATCC 49619 (for other antibiotics).

### 2.3 Bacterial identification and drug susceptibility testing results

#### 2.3.1 Colony and microscopic morphology

**FIGURE 3:**
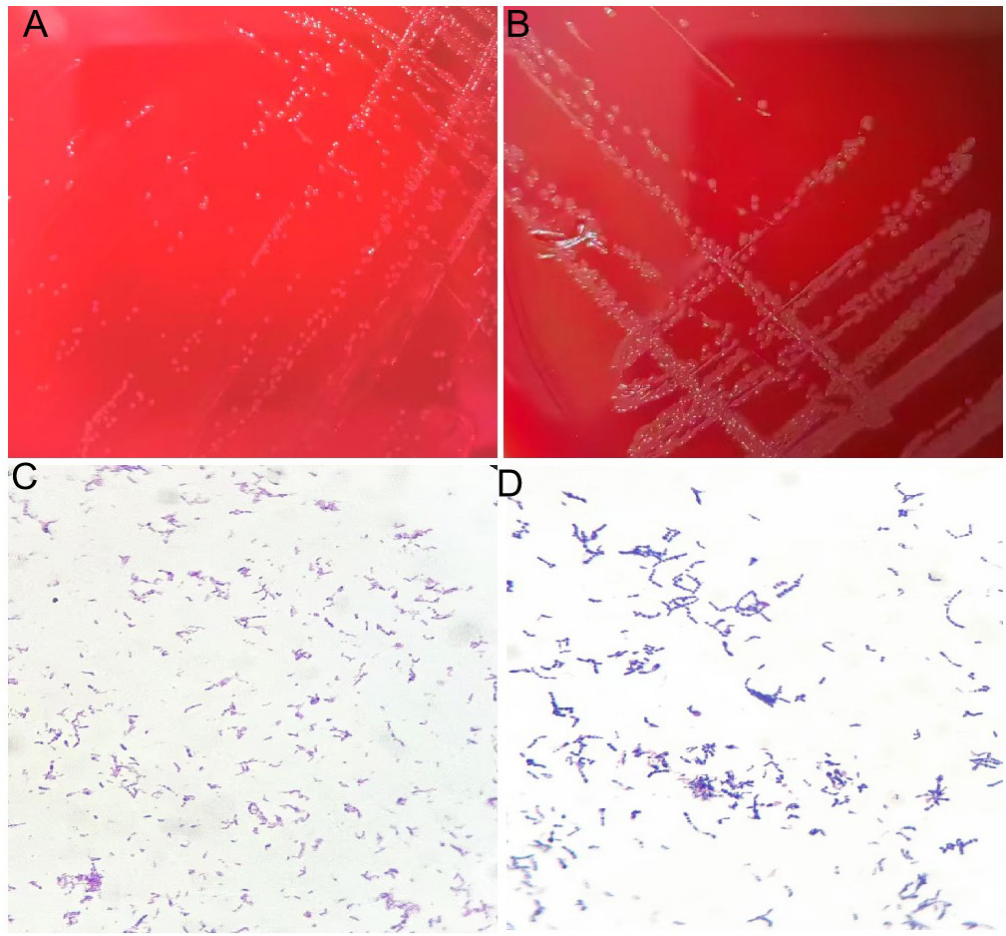
**(A)** Colonies of isolate after 72 h of culture on blood agar plate under aerobic conditions. **(B)** Colonies of isolate after 48 h of culture on blood agar plate under anaerobic conditions. **(C)** Gram staining of isolate showing gram-positive bacteria appearing as rods under aerobic conditions. **(D)** Gram staining of isolate showing gram-positive bacteria appearing as rods under anaerobic conditions.

The growth rate of the aerobic culture was relatively slow, forming small colonies with a diameter of 1 mm after 72 h of incubation. Colonies were visible on the anaerobic culture medium after 48 h of incubation. The bacteria were subjected to Gram staining and examined under a microscope, revealing Gram-positive, short bacilli. See Figure 3.

#### 2.3.2 Mass spectrometry identification

**FIGURE 4:**
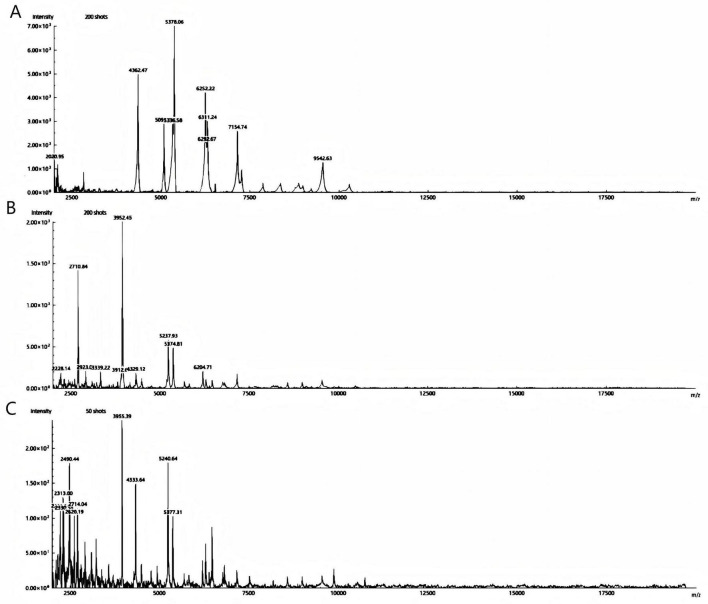
Spectrum protein peak of *Schaalia turicensis* is detected by MALDI-TOF MS; **(A,B)** aerobic and anaerobic cultured strains, respectively. **(C)** The quality control strain of *Escherichia coli* ATCC 25922.

The two bacterial strains obtained from aerobic and anaerobic cultures were identified as *Schaalia turicensis* using MALDI-TOF MS with identification scores of 2.05 and 2.11, respectively (confidence value > 2.0 indicates reliable species identification). The results of the identifications were consistent. See Figure 4. To further confirm the identification, genomic DNA from the isolate was subjected to targeted DNA sequencing by an external commercial laboratory. Sequence analysis revealed 99.6% identity with *Schaalia turicensis* strain APL10.

#### 2.3.3 Drug susceptibility testing

Table 1 shows the results of drug susceptibility testing. The MIC values presented: Penicillin (0.006 μg/mL, S), Ampicillin (0.12 μg/mL, S), Imipenem (0.12 μg/mL, S), Erythromycin (0.12 μg/mL, S), Clindamycin (256 μg/mL, R), Cefathiamidine (4 μg/mL, R), Ceftriaxone (8 μg/mL, R), Gentamicin (1 μg/mL, S).

**TABLE 1:** Drug susceptibility testing of *Schaalia turicensis* strains.

Antibiotics	*Schaalia turicensis* MIC (μg/mL)	Breakpoint values	Results
Penicillin	0.006	S ≤ 0.12,R > 4	S
Ampicillin	0.12	S ≤ 0.12,R > 4	S
Imipenem	0.12	S ≤ 4,R > 16	S
Erythromycin	0.12	S ≤ 0.5,R > 2	S
Clindamycin	256	S ≤ 0.5,R > 4	R
Cefathiamidine	4	S ≤ 8,R > 64	R
Ceftriaxone	8	S ≤ 1,R > 4	R
Gentamicin	1	S ≤ 4,R > 16	S

QC, quality control; MIC, minimum inhibitory concentration; S, susceptible; R, resistant. Breakpoints based on CLSI M45-A3; QC strains: *Escherichia coli* ATCC 25922 (gentamicin), *Streptococcus pneumoniae* ATCC 49619.

## 3 Discussion

The clinical presentation of this condition can range from being completely asymptomatic to mimicking pelvic malignancy, with symptoms such as abnormal vaginal bleeding, foul-smelling discharge, and pelvic pain, which can easily be overlooked or misdiagnosed (1, 2, 7). It has been described as one of the most frequently misdiagnosed diseases. In this case, the patient presented with abnormal vaginal bleeding and a foul-smelling discharge, which are typical manifestations of chronic endometritis. The presence of a uterine mass detected by ultrasound further complicated the diagnosis, as it could potentially be mistaken for a neoplastic lesion. However, histopathological examination ruled out malignancy, emphasizing the importance of comprehensive diagnostic evaluation in such cases.

Accurate identification of *Schaalia turicensis* remains challenging due to its slow growth and resemblance to mucosal flora (9, 10). Conventional phenotypic systems (e.g., API Rapid ID 32A) risk misidentifying it as *Gardnerella vaginalis* or *Actinomyces* spp. (11). While 16S rRNA sequencing offers reliability, cost and accessibility limit its use. In this case, MALDI-TOF MS was pivotal for species-level identification, achieving high-confidence scores (2.0) for both aerobic and anaerobic isolates—later confirmed by genomic sequencing (99.6% identity with *S. turicensis* APL10). This highlights MALDI-TOF MS as a frontline tool for fastidious organisms, though its efficacy depends on database completeness and operator expertise—limitations to consider in resource-constrained settings.

*Schaalia turicensis* is generally found in the genital and urinary tracts, as well as in infections related to the skin and intrauterine devices (IUDs) (12, 13). In women without a history of IUD use, the development of uterine actinomycosis is extremely rare. Non-IUD endometrial cases are exceptionally rare, with only three prior reports (ages 52–78) (14–16). All presented with presenting with postmenopausal bleeding (PMB) and pelvic pain; notably, 2/4 cases (including ours) had diabetes. Our patient’s 20-year history of poorly controlled diabetes (fasting glucose 8.9 mmol/L, postprandial 24.7 mmol/L) aligns with Hagiya’s case (glucose 425 mg/dL), suggesting hyperglycemia may disrupt endometrial immunity through impaired neutrophil function and microvascular compromise, facilitating bacterial invasion in atrophic endometrium. However, unlike Hagiya’s perforated pyometra requiring hysterectomy, our case manifested as chronic inflammation without complications—indicating a broader disease spectrum influenced by host factors or strain virulence. It should be particularly noted that Mirza reported a case of a 60-year-old woman who developed endometrial actinomycosis (identified only to the genus *Actinomyces* level) 10 years after IUD removal (17). Critically, this is the first MALDI-TOF MS-confirmed *Schaalia turicensis* endometrial infection without IUD association, advancing beyond genus-level identifications (*Actinomyces* spp.) in prior reports.

Empirical therapy with cefathiamidine/tinidazole failed, consistent with susceptibility testing showing resistance to cefathiamidine (MIC 4 μg/mL) and ceftriaxone (MIC 8 μg/mL)—a phenotype reported in *Schaalia* strains (18, 19). The initial choice of cefathiamidine (a first-generation cephalosporin) and tinidazole was based on empirical guidelines for pelvic infections, which target common pathogens like *Streptococcus*, *Enterococcus*, and anaerobes (7). However, this regimen did not account for potential resistance in fastidious organisms like *Schaalia turicensis*. Adjustment to amoxicillin-clavulanate (1.2 g IV q8h) resolved symptoms and reduced the uterine mass, corroborating β-lactams as cornerstone therapy (13). The optimal duration for *S. turicensis* infections follows actinomycosis guidelines: prolonged therapy (2–6 months) to prevent relapse due to the pathogen’s indolent nature (20). Here, intravenous amoxicillin-clavulanate was administered for 7 days, followed by oral amoxicillin (0.5 g q8h) for 1 month, ensuring eradication. While no formal step-down guidelines exist for *Schaalia turicensis*, oral β-lactams (e.g., amoxicillin) are preferred for continuity based on susceptibility (18). The resistance to cephalosporins but susceptibility to penicillin (MIC 0.006 μg/mL), ampicillin (MIC 0.12 μg/mL), and imipenem (MIC 0.12 μg/mL) underscores that species-level identification and susceptibility testing are imperative even for “typically susceptible” genera. Oral amoxicillin (0.5 g q8h) for 1 month ensured eradication, aligning with actinomycosis guidelines recommending prolonged therapy (20). Erythromycin susceptibility (MIC 0.12 μg/mL) offers an alternative for penicillin-allergic patients. Furthermore, source control via curettage likely contributed to treatment success by debriding infected tissue and reducing bacterial burden, synergizing with antibiotic efficacy (7). This dual approach—mechanical intervention followed by targeted antibiotics—was critical in resolving the infection.

This case challenges assumptions about *Schaalia turicensis* susceptibility. Although β-lactam resistance is uncommon, our findings and prior data confirm its possibility. Routine AST for such isolates—guided by CLSI M45-A3—is warranted, particularly when empirical therapy fails. In summary, non-IUD pelvic actinomycosis should be suspected in diabetic postmenopausal women with refractory PMB. We advocate: (1) prioritizing anaerobic cultures with extended incubation (≥5 days) for CE cases; (2) routine MALDI-TOF MS/sequencing for precise identification; (3) AST-guided therapy, noting potential cephalosporin resistance; (4) long-course β-lactams (e.g., penicillin G or amoxicillin-based regimens) as first-line.

## Data Availability

The original contributions presented in this study are included in this article/supplementary material, further inquiries can be directed to the corresponding authors.
